# Evaluating the quality of interaction between medical students and nurses in a large teaching hospital

**DOI:** 10.1186/1472-6920-6-23

**Published:** 2006-04-25

**Authors:** Gregory J Nadolski, Mary A Bell, Barbara B Brewer, Richard M Frankel, Herbert E Cushing, James J Brokaw

**Affiliations:** 1 Office of Medical Student Affairs, Indiana University School of Medicine, 635 Barnhill Drive, MS-164, Indianapolis, Indiana 46202-5120, USA; 2 Office of Professional Practice, John C Lincoln North Mountain Hospital, 250 East Dunlap Avenue, Phoenix, Arizona 85020, USA; 3 The Regenstrief Institute, Indiana University School of Medicine, 1050 Wishard Boulevard, RG-6, Indianapolis, Indiana 46202-2872, USA

## Abstract

**Background:**

Effective health care depends on multidisciplinary collaboration and teamwork, yet little is known about how well medical students and nurses interact in the hospital environment, where physicians-in-training acquire their first experiences as members of the health care team. The objective of this study was to evaluate the quality of interaction between third-year medical students and nurses during clinical rotations.

**Methods:**

We surveyed 268 Indiana University medical students and 175 nurses who worked at Indiana University Hospital, the School's chief clinical training site. The students had just completed their third year of training. The survey instrument consisted of 7 items that measured "relational coordination" among members of the health care team, and 9 items that measured psychological distress.

**Results:**

Sixty-eight medical students (25.4%) and 99 nurses (56.6%) completed the survey. The relational coordination score (ranked 1 to 5, low to high), which provides an overall measure of interaction quality, showed that medical students interacted with residents the best (4.16) and with nurses the worst (2.98; p < 0.01). Conversely, nurses interacted with other nurses the best (4.36) and with medical students the worst (2.68; p < 0.01). Regarding measures of psychological distress (ranked 0 to 4, low to high), the interpersonal sensitivity score of medical students (1.56) was significantly greater than that of nurses (1.03; p < 0.01), whereas the hostility score of nurses (0.59) was significantly greater than that of medical students (0.39; p < 0.01).

**Conclusion:**

The quality of interaction between medical students and nurses during third-year clinical rotations is poor, which suggests that medical students are not receiving the sorts of educational experiences that promote optimal physician-nurse collaboration. Medical students and nurses experience different levels of psychological distress, which may adversely impact the quality of their interaction.

## Background

The past three decades have witnessed fundamental changes in the way health care is financed and delivered in the United States and other industrialized countries. Faced with mounting economic, social, and political pressures, health services institutions have struggled to adopt methods of quality improvement to enhance both patient outcomes and cost-effectiveness. One consequence of the increased emphasis on quality has been a greater reliance on multidisciplinary collaboration and teamwork in patient care. Ideally, the two principal members of the health care team – physicians and nurses – should work together in a true partnership, marked by mutual understanding of roles and responsibilities, and shared mutually-derived clinical goals. Achieving such collaborative relationships appears to offer tangible benefits in terms of improved patient outcomes [1-3].

Although health care teams are intended to foster cooperation, the relationship between physicians and nurses is often less than optimal, even adversarial [4-6]. A variety of factors have been offered to explain the physician-nurse relationship, including medical students' misconceptions about the responsibilities and capabilities of nurses [7,8]. Laschinger and Weston [8] reported that fourth-year medical students were less knowledgeable about the competencies important for nursing than were fourth-year nursing students in their knowledge of the competencies important for medicine. This observation suggests that medical students, even those close to completing their training, do not have the sort of knowledge base about nurses that would encourage optimal interaction and lead to collaborative decision-making.

The clinical rotations in the third year of medical school might be expected to have an enormous influence on the professional development of physicians-in-training. It is during this time that medical students are inculcated – for better or worse – with the beliefs and attitudes of their physician role models, and when they experience first hand how status and hierarchy work in the health care team. Interestingly, the nature and extent of the interactions between medical students and nurses have not been systematically evaluated. In this exploratory study, we used a survey to assess medical students' perceptions about their interactions with nurses and other health care team members during the third-year clinical rotations. At the same time, the nurses on these services were surveyed about their interactions with the medical students and other health care team members. The results enabled us to gauge the medical students' and nurses' relative perceptions of how well they interacted with each other and with other members of the health care team.

## Methods

### Subjects and setting

During July and August of 2004, we contacted 268 medical students who had just completed their third year and requested that they complete an anonymous survey administered through the School's online course management system. Periodic e-mail reminders were sent during the data collection period. Students were asked to consider their third-year experiences at Indiana University Hospital, a large, multi-specialty teaching hospital that serves as the School's chief clinical training site. To provide the nurses' perspective, 175 nurses who worked on hospital services that provide core clerkship training for medical students were asked by their supervisors to anonymously complete a paper version of the survey. The nurses were asked to consider their clinical experiences in the preceding 12 months. The research was granted exempt status by the University's Institutional Review Committee.

### Survey instrument

Aside from minor modifications in wording, the surveys administered to medical students and nurses were identical and consisted of two parts. The first part was a 7-item questionnaire adopted from Gittell et al. [3] that measures "relational coordination" among members of a health care team, which, for our purposes, was considered to consist of medical students, registered nurses, resident physicians, and attending physicians. A relatively new scale, relational coordination has been validated in two service processes that require efficient interaction among workers – flight departures and patient care [3,9]. It encompasses 4 communication dimensions (frequency, timeliness, accuracy, and problem-solving communication) and 3 relationship dimensions (shared knowledge, shared goals, and mutual respect) [3]. Each of these dimensions is ranked on a 1 to 5 scale to denote the respondent's degree of interaction with other members of the health care team. The average of all 7 dimensions provides an overall measure of relational coordination between one team member and another, e.g., between medical students and nurses. In their health care setting, Gittell et al. [3] found that the overall relational coordination score had a reliability index of 0.85 (Cronbach's alpha).

The second part of the survey contained 9 items derived from the Brief Symptom Inventory (BSI), a psychological self-report symptom scale [10]. This validated scale is commonly used by mental health professionals for diagnostic purposes. The particular BSI items chosen encompass 2 symptom dimensions, interpersonal sensitivity and hostility. The interpersonal sensitivity dimension reflects feelings of personal inadequacy and inferiority, whereas the hostility dimension reflects hostile thoughts, feelings, and actions [10]. Each symptom dimension is ranked on a 0 to 4 scale of distress. We reasoned that the often-stressful environment of the wards, especially for third-year medical students, might manifest as symptoms of distress that could adversely impact the relational coordination among team members. Some studies have documented significant psychopathologies resulting from unpleasant training experiences [11]. The interpersonal sensitivity and hostility scales are reported to have reliability indices (Cronbach's alpha) of 0.74 and 0.78, respectively [10].

Also collected were basic demographic data, information about the composition of the respondent's health care team, and the names of specific hospital services on which the respondent trained or worked. Each respondent was given the opportunity to provide written comments about his or her experiences.

### Statistical analysis

Data were analyzed using SPSS version 12.0 (SPSS, Inc., Chicago, IL). Ranked responses were evaluated using Friedman's nonparametric two-way ANOVA or the Mann-Whitney U test with Bonferroni's correction. Differences between mean ranks were considered significant if p < 0.05.

## Results

### Participation rates and demographics

Of the 268 medical students invited to participate, 68 returned completed surveys (25.4%). Sixty-one of the respondents were white (89.7%) and 35 were female (51.5%); the class as a whole was approximately 86% white and 44% female. Of the 175 nurses invited to participate, 99 returned completed surveys (56.6%). The nurses were predominately white (93.9%) and female (98.0%).

### Relational coordination between medical students and nurses

Medical students and nurses interacted with one another far less effectively than they interacted with other members of the health care team (Table 1). As seen in the composite relational coordination scores, medical students interacted with residents the best (4.16) and with nurses the worst (2.98). Medical students ranked their interactions with residents as being significantly greater than their interactions with any other team member (p < 0.01 by Friedman's test). Conversely, nurses interacted with other nurses the best (4.36) and with medical students the worst (2.68). Nurses ranked their interactions with other nurses as being significantly greater than their interactions with any other team member (p < 0.01 by Friedman's test). For each category of team member, except attending physicians, the composite relational coordination score reported by medical students was significantly different from the corresponding score reported by nurses (p < 0.01 by Mann-Whitney U test).

Although the above description refers specifically to the composite relational coordination scores, similar differences were noted among the communication and relationship component scores (Table 1).

Medical students and nurses perceived the composition of their health care teams somewhat differently. In response to the phrase, "my usual care team consisted of", 100% of the medical students indicated that residents were "often" or "constantly" on their team, but only 45.6% characterized nurses as being team members. By contrast, 96.9% of the nurses indicated that other nurses were "often" or "constantly" on their team, but only 56.6% regarded medical students as having a similar presence.

### Psychological distress scores of medical students and nurses

Medical students and nurses reported different levels of psychological distress (Table 2). The interpersonal sensitivity score of medical students was significantly greater than that of nurses (1.56 versus 1.03; p < 0.01 by Mann-Whiney U test), whereas the hostility score of nurses was significantly greater than that of medical students (0.59 versus 0.39; p < 0.01 by Mann-Whitney U test).

## Discussion

Although preliminary, our findings suggest that interactions between third-year medical students and practicing nurses are suboptimal and do not provide sufficient opportunities to establish high levels of mutual understanding and collaboration. The minimal importance of nurses to medical students' concerns, coupled with the hierarchical nature of the health care team, appear to limit relationships among the team members. We speculate that neither the medical student nor the nurse views the other as being particularly relevant to fulfilling his or her role on the team. This view is supported by the relatively low relational coordination scores reported by the medical students for nurses and by the nurses for medical students.

Originally developed and validated in the airline industry, relational coordination is characterized by frequent, timely, and accurate communication, effective problem-solving, and by shared goals, shared knowledge, and mutual respect among workers [3,9]. It is intended to improve organizational performance in settings with uncertain outcomes, interdependence among workers, and significant time constraints – all hallmarks of the hospital environment. Indeed, when applied to this setting, relational coordination was associated with improved quality of care and reduced lengths of stay [3]. Relational coordination is thought to achieve these gains in efficiency by improving the exchange of information relevant to patient care. High relational coordination scores (≥ 4) suggest high levels of communication and coordination between health care providers, whereas low relational coordination scores (< 4) suggest less effective interactions.

The fact that medical students ranked residents much higher than nurses is undoubtedly a consequence of the important roles residents play in supervising medical students during their clinical rotations. A student's ultimate success or failure is closely tied to the resident's opinion of that student and his or her clinical competence. In this context, the student may strive to interact with the resident and forge a harmonious working relationship. Nurses, on the other hand, rarely if ever evaluate students or consult with students, which reduces opportunities for interaction. Students may therefore perceive nurses as having no relevance to their educational experience. This may explain the low relational coordination score reported by the medical students for nurses.

From the nurses' perspective, interacting with fellow nurses appears to be the dominant working relationship. The relational coordination score between nurses was greater than between any other pair of team members. This finding suggests that nurses exchange information and coordinate patient care with other nurses more effectively than they do with other health care providers on the team. Even the interactions with residents and attending physicians are of secondary importance. It is worth noting that nurses spend most of their time with other nurses due to the nature of their job responsibilities in health care delivery. Thus, the greater relational coordination score between nurses may partly reflect greater time spent together.

Although it is an imperfect analogy, one might think about the pilots and flight attendants on board a commercial jet as having relationships similar to physicians and nurses. Pilots and flight attendants have very circumscribed roles that largely parallel one another and involve minimal relationships. However, the performance of the entire flight crew and the flying experience of the passengers depend upon the coordinated efforts of both groups. Nurses have a lot to teach and medical students have a lot to learn to become effective medical practitioners. The current lack of interaction is a significant limitation on the educational possibilities and role modeling that accompany learning to be a doctor.

When asked about the composition of their usual health care teams, fewer than half of the medical students and a bare majority of the nurses considered the other to be a member of their team. Although it is possible that nurses could have occasionally been on teams without medical students, it is almost inconceivable that medical students could have been on teams without nurses. It is as though the medical students and nurses operate in separate spheres, each scarcely acknowledging the presence of the other.

Taken together, these findings suggest there is a real need for interprofessional teamwork education to improve physician-nurse collaborations. Third-year medical students and practicing nurses, though ostensibly on the same team, pursue their duties largely independently of one another. There is no incentive for medical students to acquaint themselves with the responsibilities and skills of nurses with respect to patient care, or for nurses to better understand the training of physicians and its influence on treatment decisions. In less than two years, third-year medical students will become resident physicians who are expected to manage patients with nursing colleagues, and yet few will have had meaningful experiences working with nurses or an adequate appreciation of their roles on the health care team.

Previous studies have shown that second-year medical students hold views of the nursing profession that are similar to the lay public [7], and that even by their fourth year, medical students still lack a sufficient understanding of the competencies important for nursing [8]. Fagin [4] has pointed out the need for early educational experiences that promote collaborative relationships between physicians and nurses. Our findings suggest that third-year clerkships are not adequate for this purpose. If medical students are to better understand the roles of nurses, we believe that additional curricular experiences will be needed.

Much has been written about the abusive experiences some medical students encounter during their clinical rotations [11-13]. Although we did not assess abuse, per se, our findings do suggest that third-year medical students have inordinate feelings of inadequacy and inferiority, which are consistent with previously reported effects of abuse [12]. Compared to published normative data [14], the interpersonal sensitivity score reported by the medical students is strongly suggestive of psychopathology for this symptom dimension. Perhaps this is not surprising given the high levels of stress and uncertainty that third-year medical students must endure as they progress through their clinical rotations. In a related context, Cochran and Hale [15] found that college students have significantly greater interpersonal sensitivity scores than the normative adult population.

By contrast, the hostility score reported by the medical students is within normal limits, which is not consistent with previously reported effects of abuse [11]. This finding suggests that, on the whole, the medical students in our sample were largely spared from the kinds of negative experiences that produce hostile feelings. Both the interpersonal sensitivity and hostility scores reported by the nurses are substantially elevated, presumably a consequence of their stressful working conditions. The fact that our administration of selected items from the Brief Symptom Inventory was non-standard precludes valid diagnostic interpretations. Nevertheless, we believe these scores provide useful measures of the psychological stressors affecting team members and their interactions. A significant divergence of scores, like those found between medical students and nurses, may contribute to the low relational coordination.

The most significant limitation of this study is the low response rate, particularly that of the students (25.4%). To what extent our respondents were representative of the class as a whole is an open question. It is possible that the 68 students who elected to complete the survey had different attitudes and experiences compared to the students who did not respond. We have no reason to suspect this is true, but the potential for bias must be considered. It is also interesting to speculate about the fact that twice as many nurses responded to the questionnaire as did medical students. One wonders if this had been a study of student-resident interactions whether the response rate would have been different. It is possible that the low response rate actually represents part of the problem we address – medical students simply do not see the relevance of a questionnaire about nurses to their interests or concerns. Short of making it a required activity, which carries its own potential for bias, it is unclear how the response rates could have been improved.

Another limitation is recall bias. Because the students and nurses were asked to consider their experiences of the preceding 12 months, the accuracy of recalled events is necessarily limited. A third limitation is the setting. Indiana University School of Medicine and its affiliated hospital are large, Midwestern institutions with educational traditions and demographic characteristics that may differ substantially from other schools in other locales. Our findings may not generalize to other student and nurse populations.

## Conclusion

Improved physician-nurse collaboration is a laudable goal, but our findings suggest that medical students are not receiving the sorts of educational experiences necessary to advance this goal. The situation is well summarized by one medical student who commented: "I feel that all participants on the health care team would benefit from more exchange and learning about each other's role early in training. Although I feel that most doctors, residents, medical students, and nurses in the hospital are working toward the same goal and have good intentions, there are gaps in communication at times that may be resolved from an earlier understanding of how to work together efficiently and effectively." We agree with these sentiments.

Effective health care depends on multidisciplinary collaboration and teamwork. We believe that patients would ultimately benefit if medical students were explicitly trained to work with nurses in a collaborative manner. As physicians, they would be better prepared to care for their patients in partnership with nursing colleagues. Medical schools should strive to incorporate interprofessional education into the curriculum.

## Competing interests

The author(s) declare that they have no competing interests.

## Authors' contributions

GJN conceived and designed study, collected and interpreted data, revised draft manuscript. MAB contributed to design of study and interpretation of data, revised draft manuscript. BBB contributed to design of study and interpretation of data, revised draft manuscript. RMF contributed to interpretation of data, revised draft manuscript. HEC contributed to design of study and interpretation of data, revised draft manuscript. JJB contributed to interpretation of data, performed statistical analysis, wrote draft manuscript. All authors read and approved the final manuscript.

## Pre-publication history

The pre-publication history for this paper can be accessed here:



## Figures and Tables

**Table 1:** Third-Year Medical Students' and Registered Nurses' Relational Coordination Scores with Different Members of the Health Care Team, Indiana University Hospital, 2003–04

		**Health Care Team Members**			
	**Survey Respondents**	**Medical Students**	**Nurses**	**Residents**	**Attendings**

Relational Coordination* (Composite Score)	**Medical Students**	3.65 (1.01)	2.98 (0.83)	4.16 (0.48)^†^	3.71 (0.71)
	**Nurses**	2.68 (1.06)	4.36 (0.72)^§^	3.77 (0.72)	3.66 (0.86)

**Relational Coordination (Communication Component)**	**Medical Students**	3.57 (1.08)	3.15 (0.92)	4.26 (0.60)^†^	3.85 (0.82)
	**Nurses**	2.74 (1.22)	4.36 (0.78)^§^	3.97 (0.76)	3.76 (0.95)

**Relational Coordination (Relationship Component)**	**Medical Students**	3.75 (1.25)	2.75 (0.90)	4.00 (0.58)^‡^	3.53 (0.76)
	**Nurses**	2.60 (1.15)	4.37 (0.82)^§^	3.50 (0.87)	3.52 (0.95)

* Relational coordination and its two components are ranked on a 1 to 5 scale, with 5 representing the best interaction; scores are expressed as mean (SD); n = 68 medical students and 99 nurses.

Friedman's test:

^†^p < 0.01, compared to all other health care team members as ranked by medical students.

^‡^p < 0.01, compared to nurses and attendings as ranked by medical students.

^§^p < 0.01, compared to all other health care team members as ranked by nurses.

**Table 2:** Third-Year Medical Students' and Registered Nurses' Psychological Distress Scores for Two Dimensions of the Brief Symptom Inventory (BSI), Indiana University Hospital, 2003–04

	BSI Dimensions*	
**Survey Respondents**	**Interpersonal Sensitivity**	**Hostility**

**Medical Students**	1.56 (0.82)^†^	0.39 (0.50)^†^
**Nurses**	1.03 (0.85)	0.59 (0.64)

* The BSI dimensions are ranked on a 0 to 4 scale, with 4 representing the most distress; scores are expressed as mean (SD); n = 68 medical students and 97 nurses.

^† ^Mann-Whitney U test, p < 0.01, compared to corresponding nurses' score.
